# Numerical simulation of local and upwind temperature stratification on flow and dispersion in a bi-dimensional street canyon

**DOI:** 10.1371/journal.pone.0305739

**Published:** 2024-06-24

**Authors:** Xiaohui Huang, Lizhen Gao

**Affiliations:** 1 College of Modern Logistics, Shanxi Vocational University of Engineering Science and Technology, Taiyuan, Shanxi, China; 2 College of Environmental Science and Engineering, Taiyuan University of Technology, Taiyuan, Shanxi, China; Southwest Jiaotong University, CHINA

## Abstract

The thermal effect mainly includes boundary temperature stratification and the local thermal effect. The combined effect of these factors on flow and dispersion in a bi-dimensional canyon was investigated by the RANS and LES methods to evaluate their performance. The results, including the flow field, turbulent kinetic energy, temperature, heat flux, pollutant concentration and fluxes, were compared with the data from wind tunnel experiments. The comparison results showed that the RANS method severely overestimated the impact of windward heating on the flow in the canyon because of the lack of simulated flow separation ability and the limitation of the Boussinesq model, leading to an incorrect flow field and an incorrect temperature and concentration. In contrast, LES performed better mainly because of its ability to simulate flow separation. LES regenerated the right vortexes, flow field and low wind velocity. LES slightly overestimates the overall temperature in the canyon because heat exchange is eliminated in LES but difficult to avoid in the experiment. The difference in the air exchange rate at the roof level between the LES and wind tunnel data was no more than 5%, and the pollutant concentration distribution of the LES was almost the same as that of the experiments. This work emphasizes that the RANS method has limited ability to simulate flow and dispersion when the thermal effect is considered even at a reduced-scale, while LES can simulate the combined effects of incoming flow temperature stratification and local thermal effects. It is therefore suggested that if computing resources are limited and the temperature difference is not large, a steady-state calculation RANS can be used. Otherwise, LES must be performed.

## 1. Introduction

The influence of thermal effects on pollutant dispersion has received much attention. The thermal effect accounts for the thermal effect caused by atmospheric boundary layer temperature stratification and that caused by local heated surfaces.

According to the temperature vertical gradient, the atmospheric boundary layer includes an isothermal boundary layer and a non-isothermal layer. In the isothermal boundary layer (i.e., neutral), there exists an approximately adiabatic potential temperature profile in which the vertical motion of fluid particles neither increases nor decreases, and there is no buoyancy effect due to temperature stratification. A non-isothermal boundary layer, including unstable (or convective) and stable boundary layers, is a common atmospheric phenomenon [1–5] and due to the buoyancy effect, the vertical motion of fluid particles is strengthened in the former but suppressed in the later and cannot be ignored. The influence of temperature stratification on pollutant dispersion has been verified through field measurements by many researchers [6–11]. While field tests are the most realistic, their costs are high and the number of measurement points is limited. Therefore, researchers have attempted to simulate thermal stratification in wind tunnels, in which unstable (stable) temperature stratification is mainly realized by heating (cooling) the floor and (or) by applying a temperature profile at the inflow. One of the earliest and most widely verified stratified wind tunnel tests was conducted by Uehara et al. [12] by controlling the incoming flow temperature, wind velocity and floor temperature to form stable, neutral and unstable conditions. Similarly, Yassin [13, 14], Kanda and Yamao [15] and Marucci and Carpentieri [16] investigated the influence of temperature stratification on dispersion around an isolated building and in an array of buildings. Similar to wind tunnel tests, temperature stratification in the numerical simulation was controlled by setting the wall temperature and /or inlet boundary (velocity and temperature profiles) in the computational domain. There are many studies in this field [17–23].

The local thermal effect is mainly due to the local temperature rise of the building surface or ground caused by solar radiation or other artificial heating. This has been confirmed in field observations, especially in summer when solar radiation is strong and the temperature difference between the building surface, ground and nearby air is large [24–31]. In wind tunnels and numerical simulations, the local thermal effect is achieved by heating or setting a certain temperature for specific surfaces (e.g., surfaces of buildings or local ground). There is also extensive literature on this subject [32–39].

For numerical simulation, there are two major approaches to model urban air flow, namely, Reynolds-Averaged Navier-Stokes (RANS) and Large Eddy Simulation (LES). RANS is more commonly employed in the fields of engineering and academia, particularly in engineering, due to its lower computational cost and acceptable level of accuracy. On the other hand, LES has achieved higher levels of accuracy, but its time requirements are prohibitive. Many researchers have compared the performances of these two methods. Typically, Tominaga and Stathopoulos examined the performance of RANS and LES for flow and concentration fields around a cube and concluded that LES can provide important information on instantaneous fluctuations in concentration, mainly due to the reproduction of unsteady flow [40]. These authors compared LES and RANS in a three-dimensional street canyon [41] and in a building array [42]; unsurprisingly, they came to essentially the same conclusion. In addition, the accuracies of the RANS and LES simulations of cross-ventilation flows for a generic isolated building [43] and a group of buildings [44] were compared. They again found that LES performs better than RANS, largely because of the reproduction of unsteady flow features, especially anisotropic velocity fluctuations. Including, but not limited to [45–47], the above studies were conducted under neutral conditions and did not involve thermal effects. In the simulation of thermal effects, Yoshie et al assessed the ability of RANS and LES to simulate non-isothermal gas dispersion flow and concluded that RANS cannot reproduce vortex shedding phenomena, leading to an overestimation of the size of the recirculation region [48]. Then, Jiang and Yoshie employed LES in a 3D urban street model in an unstable boundary layer and revealed the role of turbulence contributions to dispersion [20]. Allegrini et al validated RANS for buoyant flows in unban street canyons by setting up a 2D street canyon. The results showed that the RANS can only show the vortex structure, but it is not sufficiently accurate to predict the detailed flow field, and it is recommended to use a three-dimensional model when considering the buoyancy effect [36]. Li et al carried out LES simulations to reveal the effect of stable stratification on dispersion within street canyons via a 2D model [18]. The LES simulation of the effects of thermal stratification on tracer gas dispersion in a cavity was carried out by McMullan and Mifsud. They found that the heated windward wall could cause a multi-vortex structure within the canyon. When the walls are cooled, the evolution of the shear layer is delayed, leading to stagnant flow in the bottom part of the canyon [49]. Chew et al. compared the RANS and LES at reduced and full scales. The results showed that when the thermal effect was included, both the RANS and LES methods performed well at reduced scales. However, RANS failed at full scale, and they recommended that if RANS were to be used for full-scale simulations, they be limited to isothermal conditions [50]. However, RANS has been widely used in the study of thermal effects [51–53].

It can be seen from the literature review that (i) currently, the majority of research on wind tunnels or numerical simulations is conducted under neutral conditions, with relatively limited consideration given to thermal effects. (ii) Thermal effects include inflow temperature stratification and local thermal effects. However, previous studies focused primarily on local thermal effects, with very limited investigations into the temperature stratification of incoming flow and even rarer investigations on the consideration of both factors together. (iii) Numerous prior studies have confirmed the superiority of the LES model in reproducing flow and concentration fields under neutral conditions. Can this superiority be maintained when accounting for thermal effects? These aspects warrant further investigation. For scenarios where the wind blows vertically toward an almost infinitely long street canyon while the windward side is heated, a multi-vortex structure undoubtedly emerges. Will this multi-vortex structure change when the incoming flow also contains a temperature gradient? Can RANS or LES capture this multi-vortex structure? How do those models perform?

The innovation of this paper lies in two aspects. (i) The coupling effect between incoming temperature stratification and local thermal effects on flow and pollutant dispersion is investigated through numerical simulation for the first time. (ii) This paper presents a comparative analysis of the performance of LES and RANS models in simulating the coupling effect of thermal effects at a reduced scale, providing specific recommendations for model selection.

This paper is organized as follows: the “methodology” section describes the numerical method, wind tunnel experiments and simulation setup. The main results and discussion are introduced in Section 3, and the conclusion is presented in Section 4.

## 2. Methodology

### 2.1 Numerical methods

#### 2.1.1 RANS method

The standard *k-ε* turbulence model [54] with enhance wall function [55] of ANSYS Fluent was adopted. The turbulence kinetic energy (*k*) and its rate of dissipation (*ε*) are obtained from the following transport equations:

$$\frac{\partial }{\partial t}(\rho k)+\frac{\partial }{\partial x_{i}}(\rho ku_{i})=\frac{\partial }{\partial x_{j}}\left[(\mu +\frac{\mu_{t}}{\sigma_{k}})\frac{\partial k}{\partial x_{j}}\right]+G_{k}+G_{b}-\rho \varepsilon +S_{k}$$ (1)

$$\frac{\partial }{\partial t}(\rho \varepsilon )+\frac{\partial }{\partial x_{i}}(\rho \varepsilon u_{i})=\frac{\partial }{\partial x_{j}}\left[(\mu +\frac{\mu_{t}}{\sigma_{\varepsilon }})\frac{\partial \varepsilon }{\partial x_{j}}\right]+C_{1\varepsilon }\frac{\varepsilon }{k}(G_{k}+{C_{3\varepsilon }G}_{b})-C_{2\varepsilon }\rho \frac{\varepsilon^{2}}{k}+S_{\varepsilon }$$ (2)

$$\mu_{t}=\rho C_{\mu }\frac{k^{2}}{\varepsilon }$$ (3)

$$G_{k}=-\rho \bar{u_{i}^{\prime }}\bar{u_{j}^{\prime }}\frac{\partial u_{j}}{\partial x_{i}}$$ (4)

$$G_{b}=\beta g_{i}\frac{\mu_{t}}{{Pr}_{t}}\frac{\partial T}{\partial x_{i}}=-g_{i}\frac{\mu_{t}}{{\rho Pr}_{t}}\frac{\partial \rho }{\partial x_{i}}$$ (5)

$$\beta =-\frac{1}{\rho }{\left(\frac{\partial \rho }{\partial T}\right)}_{p}$$ (6)

Where *ρ* is the air density; *u*_*i*_ is the mean velocity component in *x*_*i*_ direction; *μ* is the molecular viscosity; *μ*_*t*_ is the turbulent viscosity computed by combining *k* and *ε* as Equ. (3), where *C*_*μ*_ is a constant (= 0.09); *G*_*k*_ represents the generation of turbulence kinetic energy due to the mean velocity gradients; *G*_*b*_ is the generation of turbulence kinetic energy due to buoyancy; *C*_1*ε*_, *C*_2*ε*_ and *C*_3*ε*_ are constants; *σ*_*k*_ and *σ*_*ε*_ are the turbulent Prandtl numbers for *k* and *ε*, equal to 1.0 and 1.3 respectively; *S*_*k*_ and *S*_*ε*_ are user-defined source terms; *Pr*_*t*_ is the turbulent Prandtl number for energy (= 0.85); *g*_*i*_ is the component of the gravitational vector in the *x*_*i*_ direction; *β* is the coefficient of thermal expansion.

Enhanced wall function is a method that can extend its applicability throughout the near-wall region (that is, viscous sub-layer, buffer region, and fully-turbulent outer region) by blending the liner (laminar) and logarithmic (turbulent) law-of-the-wall using a function suggested by Kader [56]:

$$u^{+}=e^{\Gamma }u_{lam}^{+}+e^{1/\Gamma }u_{turb}^{+}$$ (7)

where the blending function is given by:

$$\Gamma =-\frac{0.01(y^{+}{)}^{4}}{1+5y^{+}}$$ (8)

The equation for the derivative $\frac{du^{+}}{dy^{+}}$ is

$$\frac{du^{+}}{dy^{+}}=e^{\Gamma }\frac{du_{lam}^{+}}{dy^{+}}+e^{1/\Gamma }\frac{du_{turb}^{+}}{dy^{+}}$$ (9)

$$\frac{du_{lam}^{+}}{dy^{+}}=1+\alpha y^{+}$$ (10)

$$\frac{du_{turb}^{+}}{dy^{+}}=\frac{1}{\kappa y^{+}}{\left[S\prime (1-\beta u^{+}-\gamma {\left(u^{+}\right)}^{2})\right]}^{1/2}$$ (11)

$$S\prime =\left\{ \begin{array}{c} 1+\alpha y^{+},\;y^{+}<y_{s}^{+}\\ 1+\alpha y_{s}^{+},\;y^{+}\geq y_{s}^{+} \end{array}\right.$$ (12)

$$\alpha \equiv \frac{v_{w}}{\tau_{w}u^{*}}\frac{dp}{dx}=\frac{u}{\rho^{2}{\left(u^{*}\right)}^{3}}\frac{dp}{dx}$$ (13)

$$\beta \equiv \frac{\sigma_{t}q_{w}u^{*}}{C_{p}\tau_{w}T_{w}}=\frac{\sigma_{t}q_{w}}{{\rho C}_{p}u^{*}T_{w}}$$ (14)

$$\gamma \equiv \frac{\sigma_{t}{\left(u^{*}\right)}^{2}}{2C_{p}T_{w}}$$ (15)

Enhanced thermal wall function follow the same approach developed for the profile of *u*^+^:

$$T^{+}=e^{\Gamma }T_{lam}^{+}+e^{1/\Gamma }T_{turb}^{+}$$ (16)

where the blending function is defined as

$$\Gamma =-\frac{0.01(Pry^{+}{)}^{4}}{1+5{Pr}^{3}y^{+}}$$ (17)

The definition for turbulent and laminar thermal wall functions are

$$T_{lam}^{+}=Pr(u_{lam}^{+}+\frac{\rho u^{*}}{2q}u^{2})$$ (18)

$$T_{turb}^{+}=Pr\left\{u_{turb}^{+}+P+\frac{\rho u^{*}}{2q}[u^{2}+(\frac{Pr}{{Pr}_{t}}-1){\left(u_{c}^{+}\right)}^{2}{\left(u_{*}\right)}^{2}]\right\}$$ (19)

where *Pr* is the molecular Prandtl number, $u_{c}^{+}$ is the value of *u*^+^ at the fictitious crossover between the laminar and turbulent region.

#### 2.1.2 LES method

The subgrid-scale turbulence model used the Wall-Adapting Local Eddy-Viscosity (WALE) model [57], the eddy viscosity is model by

$$\mu_{t}=\rho L_{S}^{2}\frac{{\left(S_{ij}^{d}S_{ij}^{d}\right)}^{3/2}}{{\left(\bar{S_{ij}}\bar{S_{ij}}\right)}^{5/2}+{\left(S_{ij}^{d}S_{ij}^{d}\right)}^{5/4}}$$ (20)

Where *L*_*S*_ and $S_{ij}^{d}$ are defined, respectively, as

$$L_{S}=min\;(\kappa d,0.325V^{1/3})$$ (21)

$$S_{ij}^{d}=\frac{1}{2}({\bar{g_{ij}}}^{2}+{\bar{g_{ji}}}^{2})-\frac{1}{3}\delta_{ij}{\bar{g_{kk}}}^{2},\bar{g_{ji}}=\frac{\partial \bar{u_{i}}}{\partial x_{j}}$$ (22)

### 2.2 Wind tunnel experiments

The experiments were carried out in the EnFlo wind tunnel of the University of Surrey and all the data are fully available [58]. The street canyon model was an isolated bi-dimensional street canyon with height (*H*) of 166 mm, width (*W*) of 166 mm and length (*L*) of 2490 mm. A gas source (C_3_H_8_: 1.8%; diameter: 22 mm; flow rate: *Q*_*V*_ = 0.46 L/min) was on the ground in the center of the canyon. In the experiment, two kinds of approaching flow temperature stratification (neutral and stable) and five kinds of local heating configurations were investigated. Here, only the windward wall heating case with neutral and stable approaching flow were selected as the validation data since the multi-vortex structure were found in the experiment.

Two non-dimensional numbers are defined as inflow Richardson number (*Ri*_*inflow*_) to represent inflow temperature stratification and local Richardson number (*Ri*_*local*_) to represent local thermal effect. They are expressed by Eqs (23) and (24).


$${Ri}_{inflow}=\frac{g(T_{2H}-T_{0})H}{T_{0}U_{2H}^{2}}$$ (23)

$${Ri}_{local}=\frac{g(T_{2H}-T_{local})H}{T_{2H}U_{2H}^{2}}$$ (24)

In Eqs (23) and (24), *g* is the acceleration of gravity (= 9.8 m/s^2^), *T*_2*H*_ is the air temperature at *z* = 2*H*, *T*_0_ is the reference temperature, *T*_*local*_ is the temperature of the local heating wall, *U*_2*H*_ is the reference velocity at *z* = 2*H*.

Table 1 lists the inflow parameters and the local temperature settings in wind tunnel experiments.

**Table 1:** Inflow parameters and local temperature setting in wind tunnel.

Inflow parameters			Local temperament setting	
Neutral	*U*_2*H*_(m/s)	0.65	*T*_*local*_(K)	391.5
	*T*_0_(K)	297	*Ri*_*local*_	-1.27
	*T*_2*H*_(K)	297		
	*Ri*_*inflow*_	0		
Stable	*U*_2*H*_(m/s)	0.65	*T*_*local*_(K)	391
	*T*_0_(K)	292	*Ri*_*local*_	-1.18
	*T*_2*H*_(K)	298		
	*Ri*_*inflow*_	0.08		

### 2.3 CFD calculation conditions

#### 2.3.1 Computational domain and mesh resolution

The size of the street canyon and the source were consistent with those in the wind tunnel. The domain was set to 18 *H* × 15 *H* × 9 *H* (length × width × height, the width and height of the domain were consistent with the measured cross-sections in the wind tunnel test, and the upstream and downstream lengths were 5 *H* and 10 *H*, respectively).

**Fig 1:**
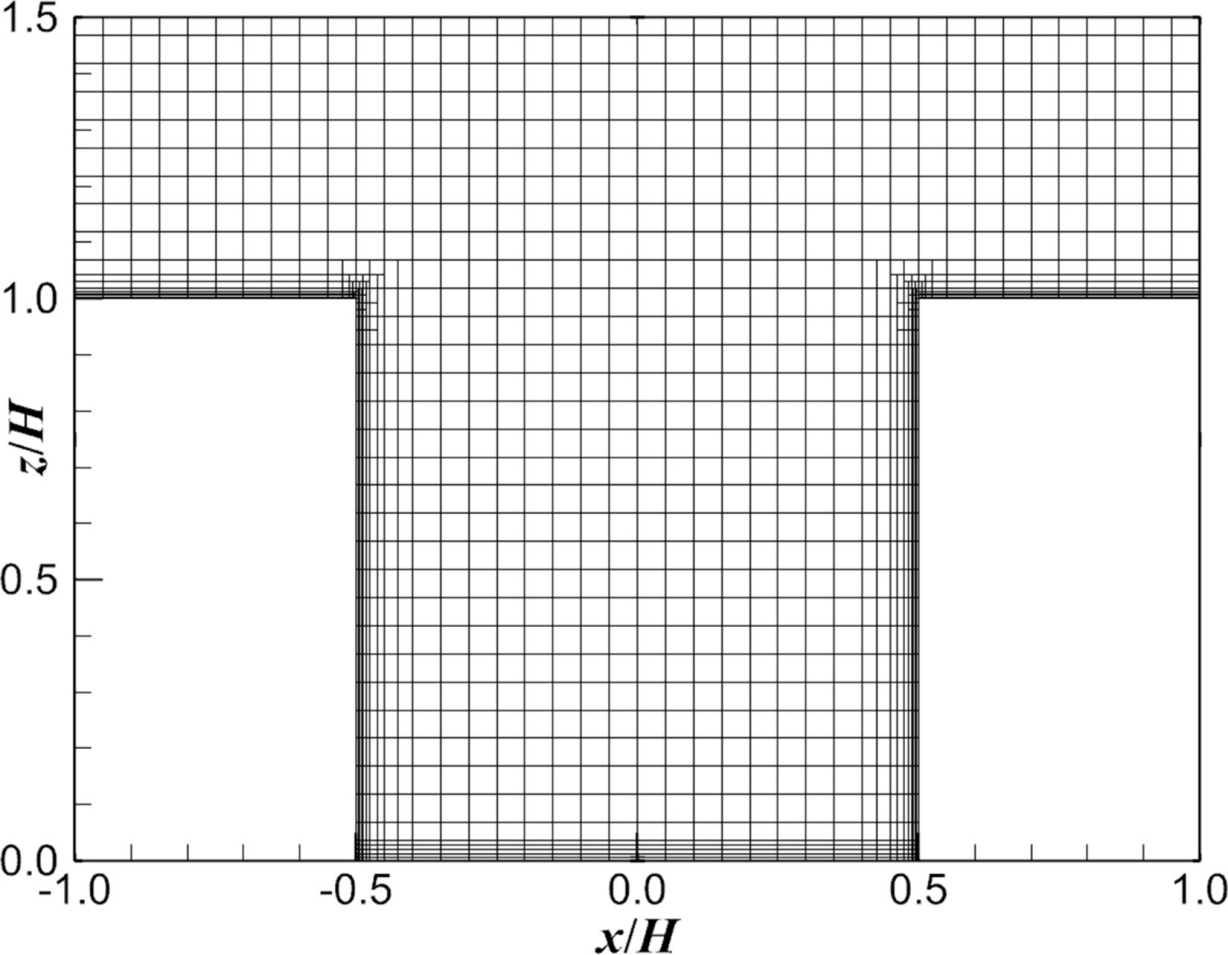
Grid system in the canyon.

Fig 1 shows the grid system in the canyon. The origin is located in the center of the source at ground level, *x* is the direction of the incoming wind, *y* is the direction perpendicular to the wind, and *z* is the distance from the ground. In this study, a nested zone and a uniform structured grid system were used. The grid size near the canyon was 0.05 *H* in the *x* direction and *z* direction and 0.1 *H* in the *y* direction. In addition, all the near-wall cells that were refined with the smallest cells had a cell size of 0.003 *H* (0.5 mm), so the *y*^+^ of all the walls was less than 5 (for this study, most of the near-wall *y*^+^ values were less than 1). The total number of grids was 0.65 million.

#### 2.3.2 Boundary conditions and solution method

1. *RANS method*. The inflow surface was set as a velocity inlet condition with the inflow profiles (including the inflow wind velocity, temperature and turbulent kinetic energy) consistent with the wind tunnel test [58]. The source was set as the mass-flow-inlet and the emission parameters were consistent with those used in the experiments. The canyon walls and the ground were set as no-slip wall boundary conditions with the temperature in line with the wind tunnel experiments, and enhanced wall functions were applied. The two sides of the domain (parallel to the *x*-axis) and the top surface were set as symmetric boundary conditions. The outlet was a pressure outlet.
  In the calculation, the Semi-Implicit Method for Pressure Linked Equations-Consistent (SIMPLEC) was used for pressure-velocity coupling, and the advection scheme was a second-order upwind difference scheme (UDS). To ensure the convergence of the calculation results, the residuals of all variables should be less than 10^−4^.
2. *LES*. For the LES, the boundary conditions were the same as those of the RANS except that the inflow turbulence was generated by the vortex method [59] at the inflow surface. In the calculation, the Bounded Central Differencing (BCD) scheme was used for convection terms. The Bounded Second Order Implicit scheme was used for transient formulation. The time step size was fixed at 0.01 *H*/*U*_2*H*_ (0.0026 s). The average value was statistically significant after 100 *H*/*U*_2*H*_ (26 s), and the averaging period was 100 *H*/*U*_2*H*_ (26 s, that is, from 26 s to 52 s). The Courant numbers were less than 1. The tolerance settings were the same as those for RANS.

## 3. Results and discussion

### 3.1 Flow

**Fig 2:**
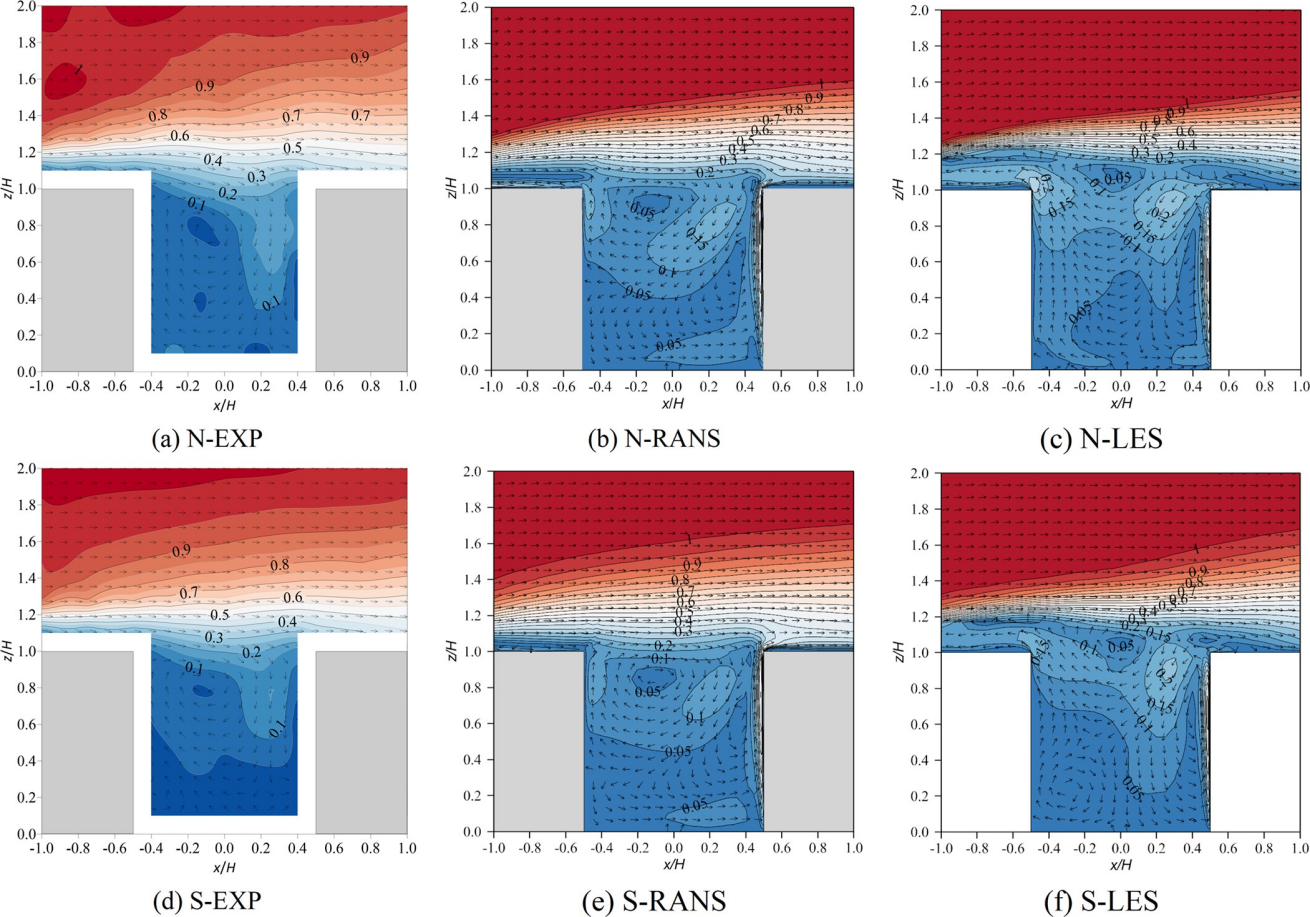
Wind vector and contour of normalized velocity of the vertical plane (*y*/*H* = 0). N represents neutral inflow condition, S represents stable inflow condition, EXP means wind tunnel experiments.

Fig 2 shows the wind vector and the contours of the normalized mean velocity ($\sqrt{u^{2}+w^{2}}/U_{2H}$) of the vertical plane (*y*/*H* = 0). It is noted that *u* and *w* are the components of the wind velocity in the *x* and *z* directions, respectively. In wind tunnel experiments, in the case of neutral inflow conditions, the buoyancy generated by local heating on the windward side was opposite to the descending motion of the air into the canyon, which slowed the velocity and produced counter-rotating double vortexes. Under stable inflow conditions, the velocity within the canyon further decreased, especially in the lower part of the canyon, and even a third counterclockwise vortex formed near the ground on the leeward side. Despite the large local Richardson number (*Ri*_*local*_ = -1.18) and inherent instability in the canyon, the stable temperature stratification of the inflow effectively impedes airflow within the canyon, particularly in its lower section (Fig 2(D)).

The RANS method overestimated the impact of windward heating on the flow in the canyon because the results showed a similar size of counter-rotating vortexes under both neutral and stable inflow conditions (Fig 2(B) and 2(E)). In addition, the velocity in the canyon simulated with RANS was slightly lower under stable conditions than under neutral conditions, but the third counter-rotating vortex was not reproduced (Fig 2(E)). Therefore, RANS cannot well simulate inflow temperature stratification. In contrast, LES performed better than RANS in terms of both the number of regenerated vortexes and the low wind velocity in the canyon, and their results were closer to the wind tunnel results (Fig 2(C) and 2(F)). However, the third eddy simulated by LES was larger than that in the experiment because the inflow air temperature in the wind tunnel was higher than that in the laboratory, and the possible heat exchange between them was excluded from the numerical simulation. It can be predicted that the inflow temperature stratification predicted by LES was more obvious than that in the experiment.

**Fig 3:**
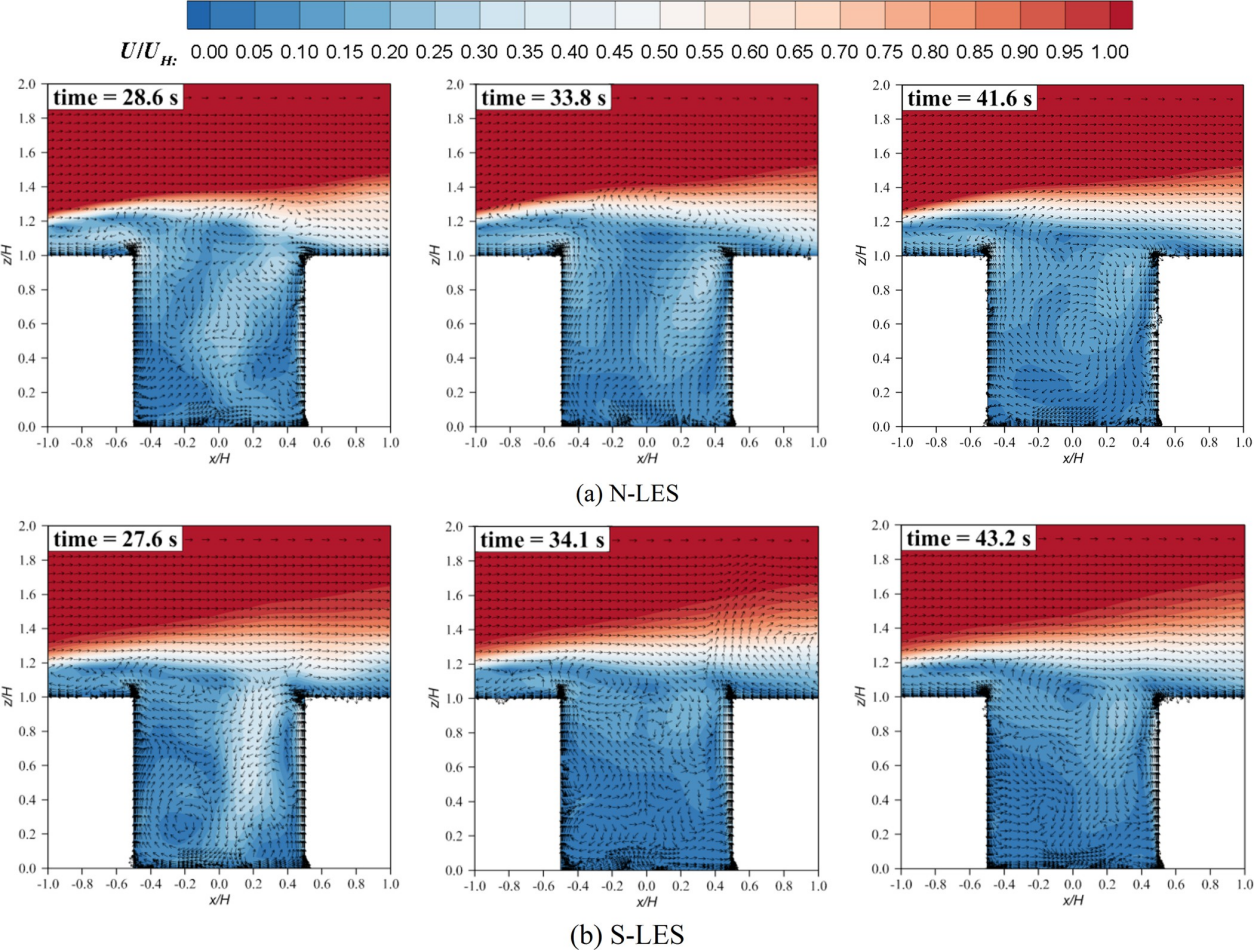
Instantaneous velocity contours and vectors at different times of the vertical plane (y/H = 0) from LES.

Why does RANS fail to predict the flow field when it encounters thermal effects? There are two hypotheses: flow separation and limitation of the Boussinesq model. First, the strong buoyant effect (updraft) driven by the heated windward wall opposes the mechanical effect (downdraft) from the freestream wind. This opposition causes flow separations. It is obvious that the steady-state solver RANS cannot reproduce such unsteady flow feature. However the transient-state solver LES can simulate vortexes within a canyon relatively accurately, mainly because LES can simulate flow separation, which has been confirmed by many researchers under neutral conditions [40–47] or non-neutral conditions [18, 20, 48–50]. The performance of LES remains satisfactory even when accounting for both inlet temperature stratification and local thermal effects. Fig 3 shows instantaneous flow fields at three different moments. For the neutral inflow condition, at 28.6 s, the strong downward flow hit against the upward buoyant flow near the windward side. At 33.8 s, on the windward wall or even in the entire canyon, buoyancy-driven updrafts clearly dominate. At 41.6 s, the strong downdrafts dominate again, with buoyancy-driven flow occurring upward only near the lower part of the windward wall. In the stable inflow case, the strength of the buoyancy-driven flow and mechanical-driven flow changes with time, and flow separation occurs in the canyon, leading to the multi-vortex structure of the mean flow field.

Another reason is the limitation of the Boussinesq model. When heat is added to a fluid and the fluid density varies with temperature, a flow can be induced due to the force of gravity acting on the density variations. The importance of buoyancy forces in a mixed convection flow can be measured by the ratio of the Grashof and Reynolds numbers: $\frac{Gr}{{Re}^{2}}=\frac{g\beta \Delta TL}{\vartheta^{2}}$. When this number approaches or exceeds unity, strong buoyancy contributes to the flow. Conversely, if it is very small, buoyancy forces may be ignored. For many natural convection flows, including those in this study, one can achieve faster convergence with the Boussinesq model than by setting up the problem with fluid density as a function of temperature. This model treats density as a constant value in all solved equations, except for the buoyancy term in the momentum equation: $(\rho -\rho_{0})g\approx -\rho_{0}\beta (T-T_{0})g$, where *ρ*_0_ is the density of the flow (a constant), *T*_0_ is the operating temperature, and *β* is the thermal expansion coefficient. This model is obtained by using the Boussinesq approximation $\rho =\rho_{0}(1-\beta \Delta T)$ to eliminate *ρ* from the buoyancy term. This approximation is accurate when density changes are small, that is, when *β*(*T*−*T*_0_)≪1. The thermal expansion coefficient of air at normal temperature and pressure is 0.00367/°C, which means that Δ*T*≪272°C. This indicates that the temperature difference must be at least one order of magnitude smaller than 272°C; that is, the temperature difference must be less than 27.2°C. However, the temperature difference in this paper was 93–99°C, which was more than three times that of 27.2°C. The consequence was that the turbulence generated by the heated wall neglected the horizontal gradients because of the inappropriate Boussinesq approximation, which was actually significant near the heated wall.

The above analysis shows that in regard to thermal effects, especially in regard to skimming flow, one must model the flow in one of the following ways: (1) Perform a steady-state calculation using the Boussinesq model, except when the temperature difference in the domain is large. As a recommendation, if the margin of error is 10%, the temperature difference should be less than 27.2°C; if the margin of error is 5%, the temperature difference should be less than 13.6°C. (2) If the temperature differences are large, it is better to perform a transient calculation, such as LES. LES more easily catches the flow separation and does not miss the turbulence caused by buoyancy.

### 3.2 Turbulent kinetic energy

**Fig 4:**
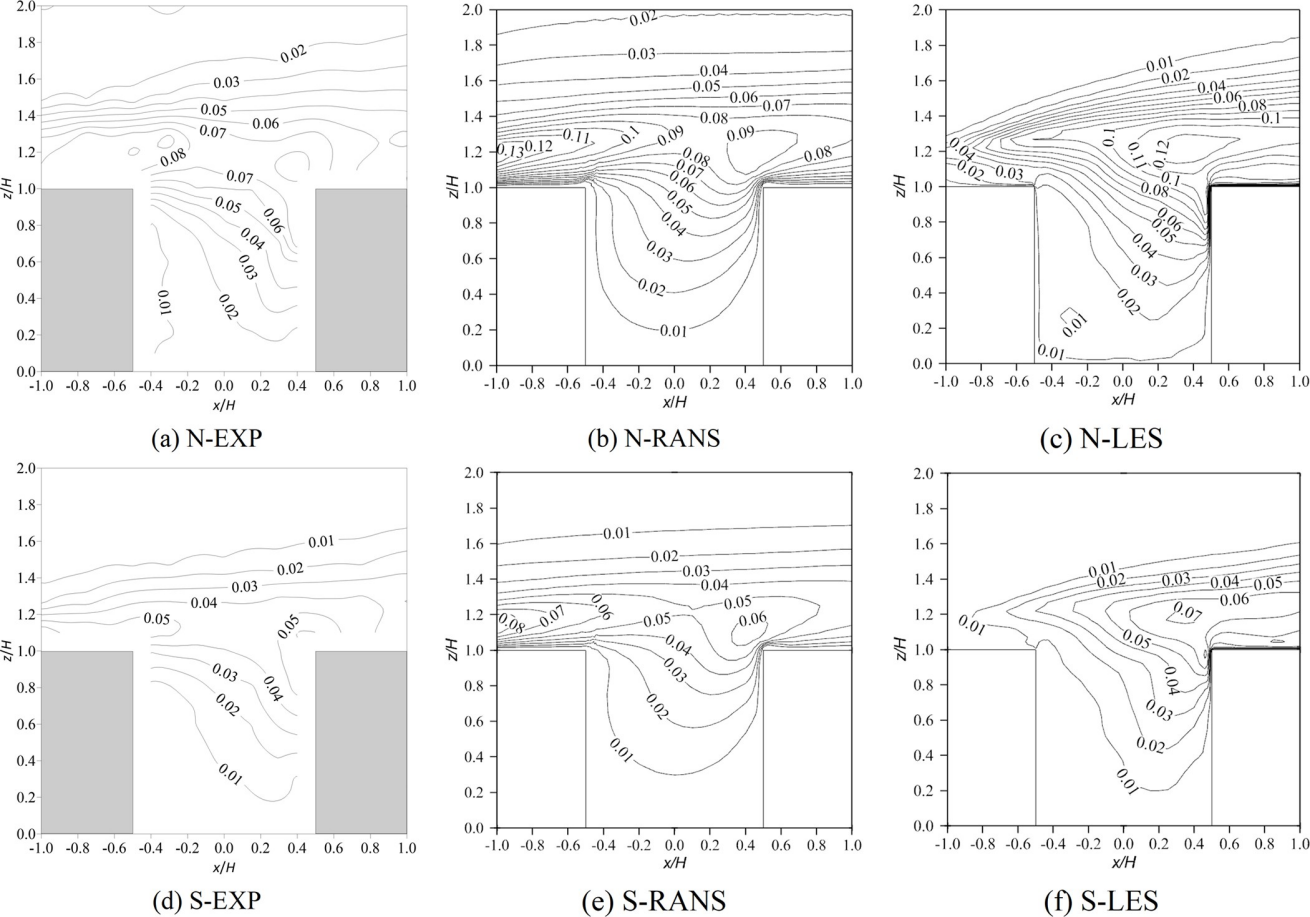
Contour of normalized turbulence kinetic energy ($k/U_{2H}^{2}$) of the vertical plane (y/H = 0). N represents neutral inflow condition, S represents stable inflow condition, EXP means wind tunnel experiments.

Fig 4 shows the distribution of the normalized turbulent kinetic energy ($k/U_{2H}^{2}$) of the vertical plane (*y*/*H* = 0). In the wind tunnel experiments, the maximum value of $k/U_{2H}^{2}$ occurred at the top of the upstream building, at 0.13 and 0.08 under neutral and stable conditions, respectively (Fig 4(A) and 4(D)). As analyzed in the above section, due to the large difference between the flow structure simulated by RANS and the wind tunnel test, the predicted $k/U_{2H}^{2}$ distribution was also significantly different, which was mainly reflected in two aspects: (i) the predicted $k/U_{2H}^{2}$ near the windward side was smaller, indicating that the turbulent kinetic energy generated by local heating could not be reflected. As explained in the previous section, RANS cannot capture the turbulence generated by heated walls because of the inappropriate Boussinesq approximation; (ii) the increasing mode of the values in the canyon was inconsistent with the wind tunnel test. In contrast, according to the LES results, the maximum values of $k/U_{2H}^{2}$ occurred at the windward corner of the downstream building and were 0.12 and 0.07 under neutral and stable conditions, respectively. In addition, LES performed better than RANS inside the canyon; in particular, the large turbulent kinetic energy value near the windward wall was reproduced well, and the $k/U_{2H}^{2}$ distribution in the canyon was almost consistent with the wind tunnel experiments.

### 3.3 Temperature and heat flux

**Fig 5:**
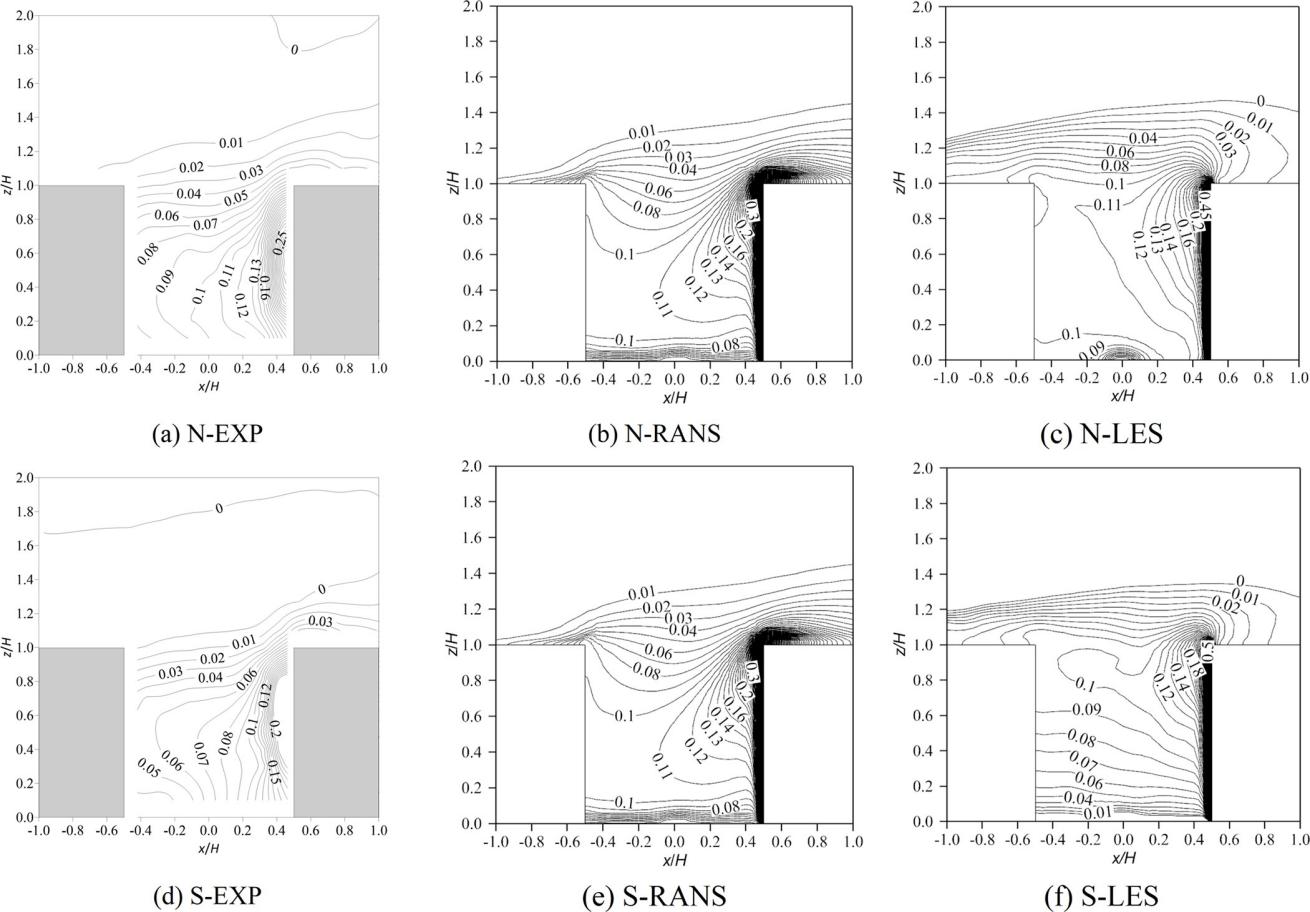
Contour of normalized temperature ($\frac{T-T_{2H}}{T_{local}-T_{2H}}$) of the vertical plane (*y*/*H* = 0). N represents neutral inflow condition, S represents stable inflow condition, EXP means wind tunnel experiments.

Fig 5 is the distribution of the normalized temperature ($\frac{T-T_{2H}}{T_{local}-T_{2H}}$) in the vertical plane (*y*/*H* = 0). In the wind tunnel experiments, the local thermal effect was mainly confined to the windward wall. The average temperature in the canyon under stable inflow conditions was slightly lower than that under neutral conditions. The maximum temperature measured in the experiment was approximately at the height of the center of the windward wall due to the buoyancy driving flow in the opposite direction to the downdraft entering the canyon. Except near the windward wall, the temperature distribution of other areas in the canyon was mainly affected by airflow movement. As shown in Section 3.1, the large difference between the flow field simulated by RANS and the wind tunnel test resulted in a difference in the temperature distribution. In addition, by comparing the N-RANS case and S-RANS case (Fig 5(B) and 5(E)), it was found that the impact of inflow temperature stratification was almost not reflected, which was consistent with the conclusion of the flow field. LES seemed to perform better than RANS but overestimated the overall temperature in the canyon. The highest temperature simulated by LES was located slightly below the windward corner. In particular, the temperature gradient in the lower part of the canyon was almost vertical under inflow stable conditions according to LES, which is significantly different from the horizontal temperature gradient in the experiment (see Fig 4(D) and 4(f)). This may be due to the use of cold water on the leeward wall and the canyon ground during the experiment, which inevitably led to heat exchange.

**Fig 6:**
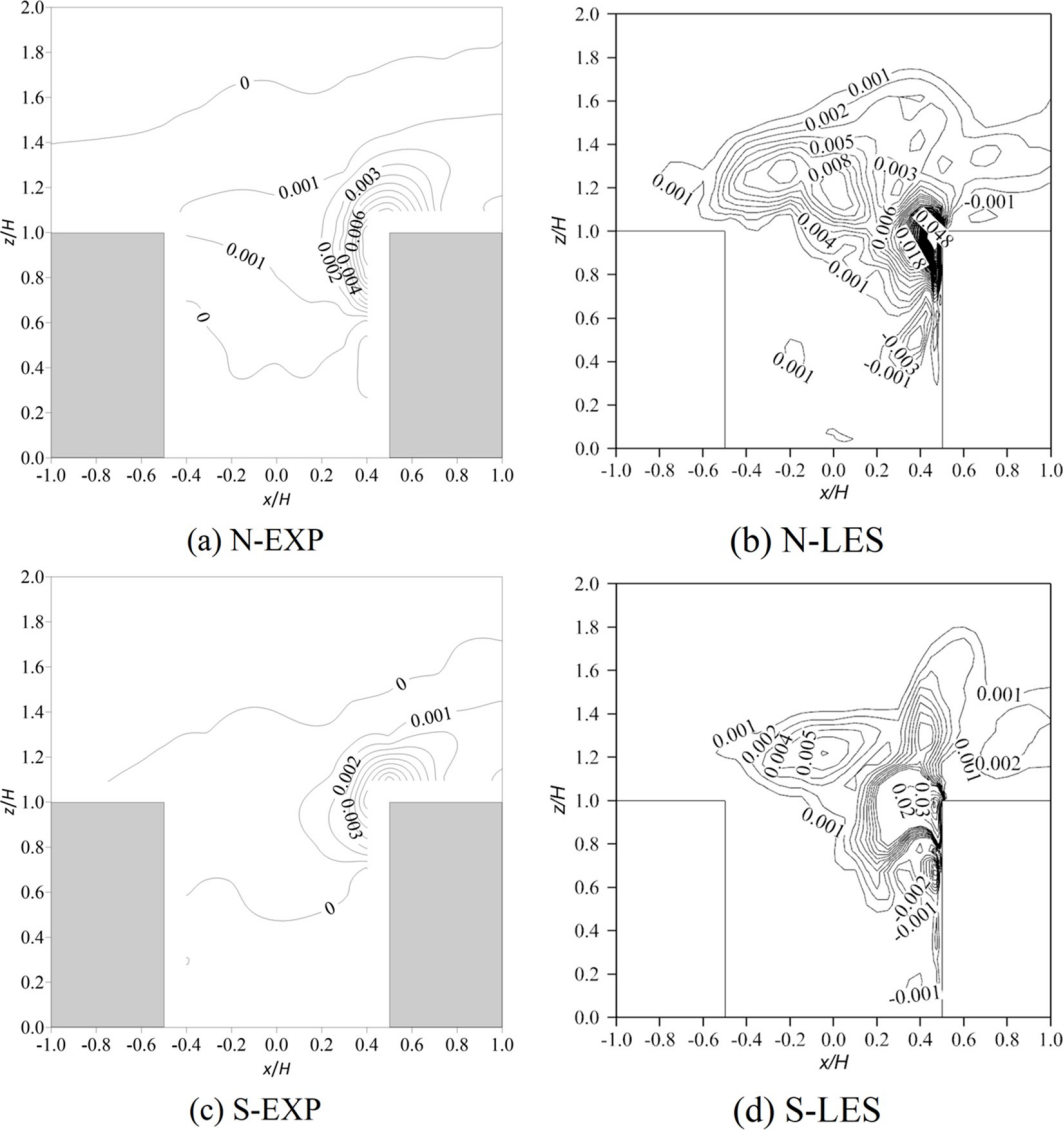
Contour of normalized turbulent vertical heat flux ($\frac{\bar{w^{\prime }T^{\prime }}}{U_{2H}(T_{local}-T_{2H})}$) of the vertical plane (y/H = 0). N represents neutral inflow condition, S represents stable inflow condition, EXP means wind tunnel experiments.

Fig 6 exhibits the distribution of the normalized turbulent vertical heat flux ($\frac{\bar{w^{\prime }T^{\prime }}}{U_{2H}(T_{local}-T_{2H}}$) in the vertical plane (*y*/*H* = 0). *w*′ and *T*′ are the fluctuations of vertical velocity and temperature, respectively. Due to the failure of the RANS simulation of the temperature distribution, only a comparison of the LES and experimental results is shown here. In the wind tunnel experiments, the peak value occurred near the windward corner, and the windward wall heating mainly affected the upper part of the canyon. Compared with the neutral inflow condition, the effect region was smaller, and the heat flux was lower, resulting in a lower temperature in the canyon (see Fig 5(D)). The LES results were generally consistent with the wind tunnel results and could distinguish the difference in inflow temperature stratification, but the thermal flux value was overestimated, which explained the overestimation of the temperature in the canyon. As mentioned earlier, heat exchange is inevitable in wind tunnel experiment, but it can be avoided in numerical simulation.

### 3.4 Pollutant concentration and fluxes

**Fig 7:**
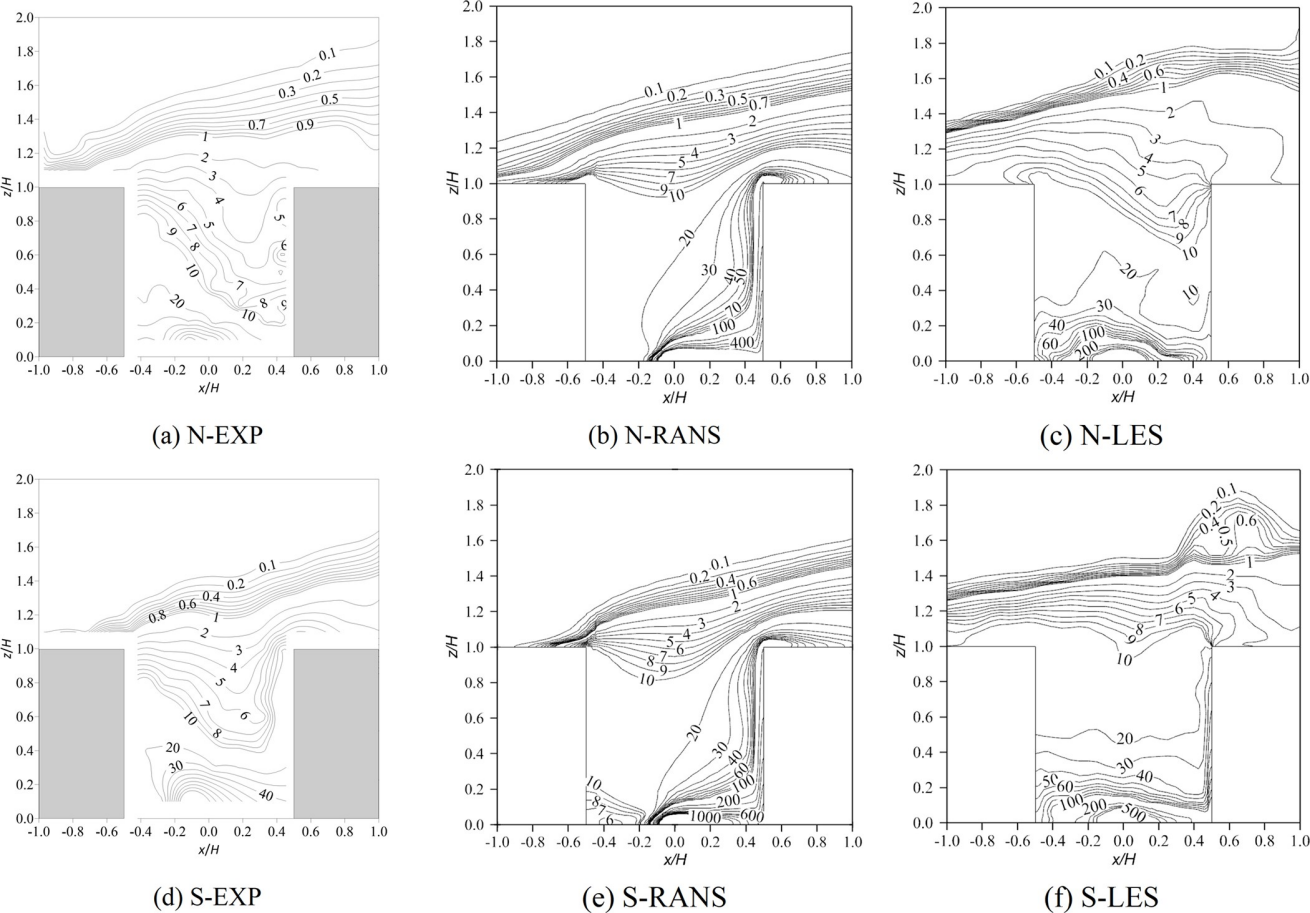
Contour of normalized concentration ($\frac{C}{C_{0}}$) of the vertical plane (y/H = 0). N represents neutral inflow condition, S represents stable inflow condition, EXP means wind tunnel experiments.

Fig 7 shows the normalized pollutant concentration ($\frac{C}{C_{0}}$) of the vertical plane (*y*/*H* = 0). *C* is the pollutant concentration, *C*_0_ is the reference concentration which is defined as $C_{0}=\frac{Q_{v}}{U_{2H}H^{2}}$, and *Q*_*v*_ is the gas flow rate. In the wind tunnel experiments, under neutral inflow conditions, pollutants were concentrated near the ground between the source and the leeward wall, and the pollutant concentration near the leeward wall was greater than that near the windward wall. Under stable inflow conditions, the overall distribution pattern was similar to that in the neutral case but with a higher concentration, except that the concentration of the corner between the leeward wall and ground was lower. Unfortunately, the RANS method failed to predict the concentration in the canyon regardless of whether the incoming flow temperature stratification was neutral or stable. A high concentration area appeared between the source and the windward wall, and the concentration on the windward wall was significantly greater than that on the leeward wall, which was completely different from the experimental result. It also indicated that the correct flow field was the basis of concentration prediction. Compared with the wind tunnel results, the LES results were better than the RANS results but still overestimated the concentrations. Near the ground of the canyon, LES predicted a nearly symmetrical distribution of concentrations along the source center (*x*/*H* = 0, *y*/*H* = 0), especially under stable inflow conditions. As mentioned in Section 3.1, LES may overestimate the thermal effect, or it may be that heat exchange in the experiment was difficult to avoid completely. As a result, the main thermal effect vortex was larger as predicted by the LES than in the experiments (see Fig 2).

**Fig 8:**
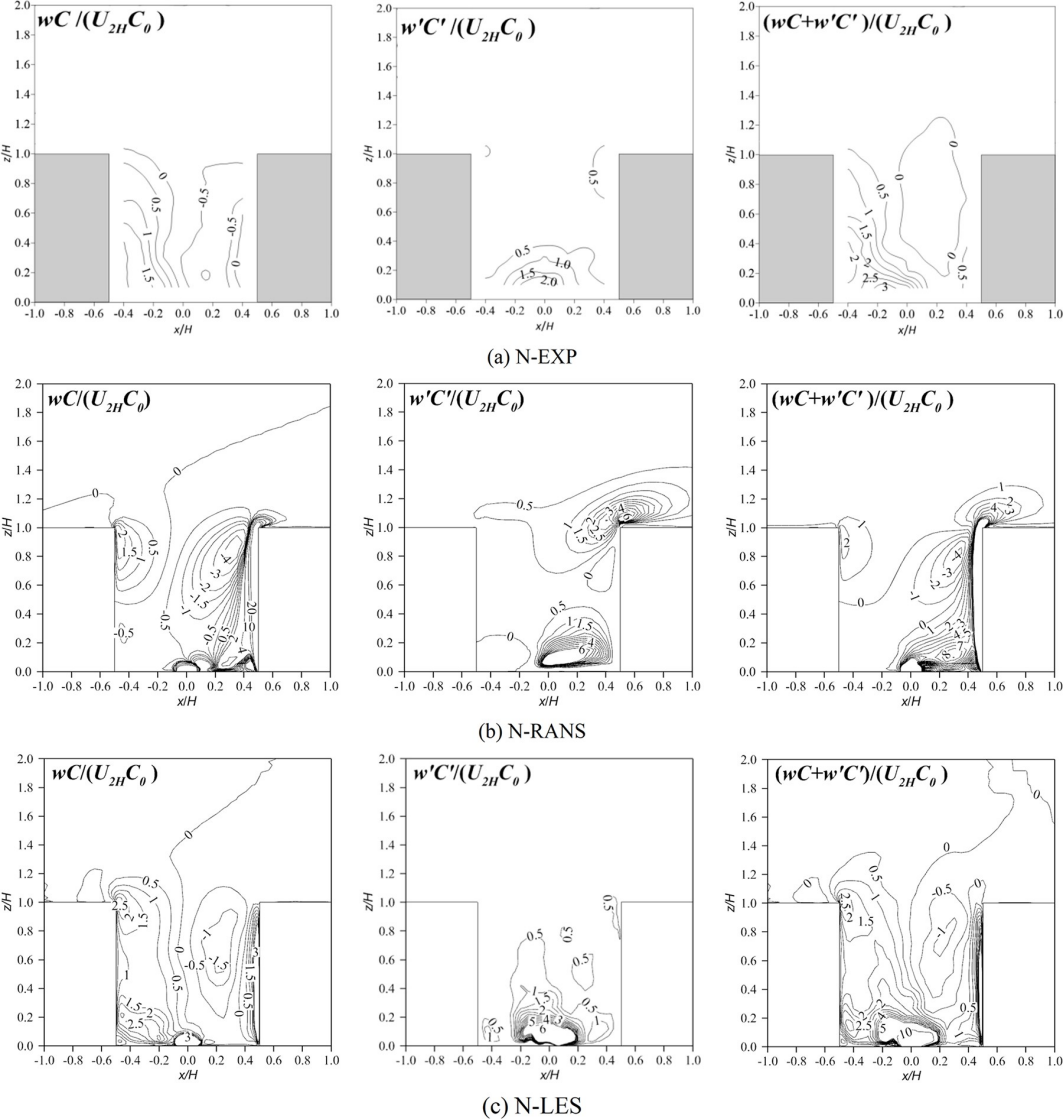
Contour of normalized vertical pollutant fluxes of the vertical plane (*y*/*H* = 0) under neutral inflow condition. N represents neutral inflow condition, S represents stable inflow condition, EXP means wind tunnel experiments.

**Fig 9:**
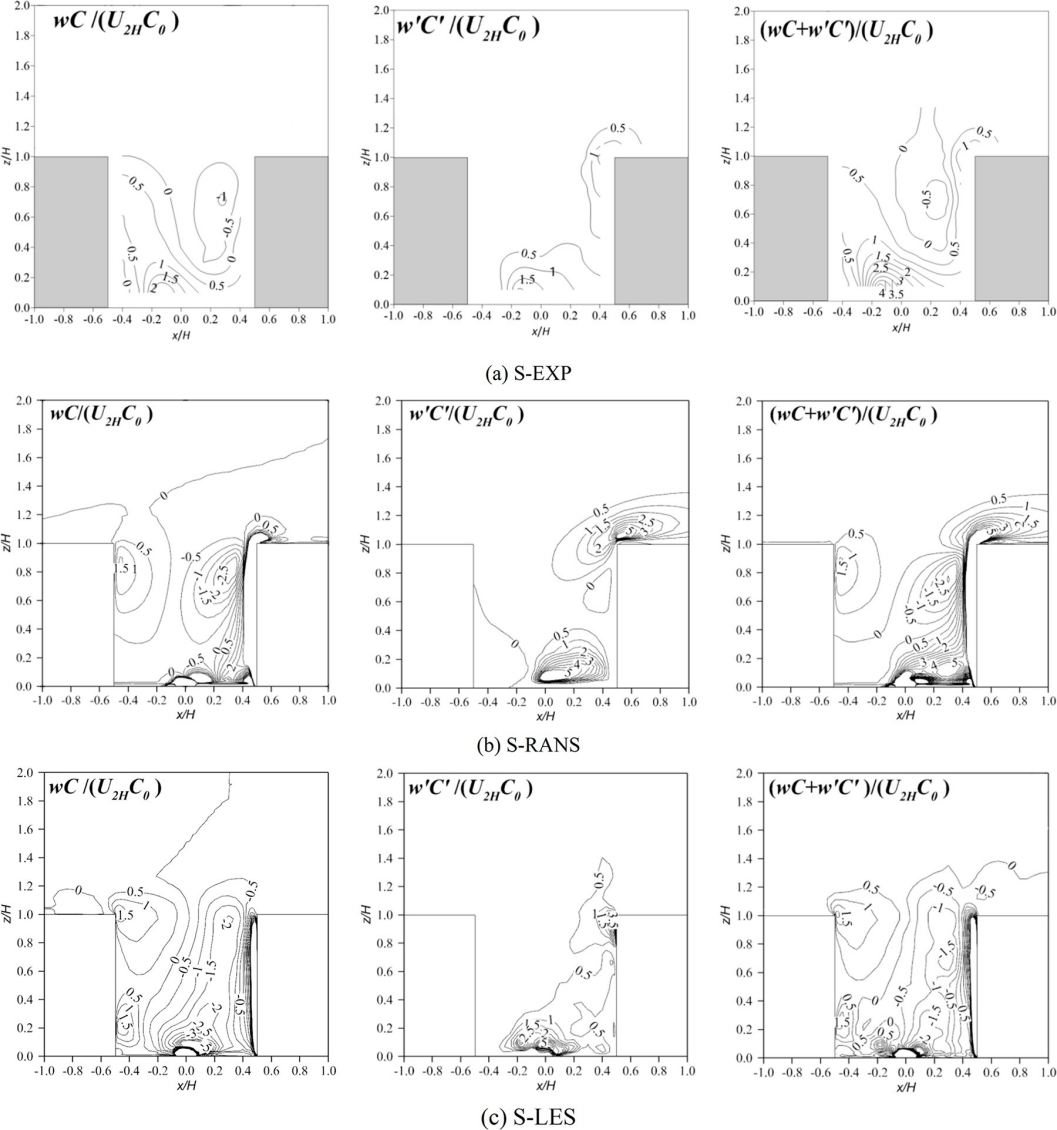
Contour of normalized vertical pollutant fluxes of the vertical plane (*y*/*H* = 0) under stable inflow condition. N represents neutral inflow condition, S represents stable inflow condition, EXP means wind tunnel experiments.

To investigate the pollutant transport mechanism, the vertical normalized flux distribution of pollutants are shown in Figs 8 and 9. *w* is the mean component of the wind velocity in the *z* direction, *w*′ is the fluctuation in the vertical velocity, *C* is the pollutant concentration, and *C*_0_ is the reference concentration. *wC*/(*U*_2*H*_*C*_0_) represents the normalized convective fluxes, *w*′*C*′/(*U*_2*H*_*C*_0_) represents the normalized turbulent fluxes, and the sum of the two $\left(w^{\prime }C^{\prime }+wC)/(U_{2H}C_{0}\right)$ is the total pollutant fluxe. In the RANS model, the turbulent fluxes are modeled by the gradient diffusion hypothesis, that is, $w^{\prime }C^{\prime }=-\frac{\vartheta_{t}}{{Sc}_{t}}\frac{\partial C}{\partial z}$, where *ϑ*_*t*_ is the eddy viscosity, and *Sc*_*t*_ is the turbulent Schmidt number (*Sc*_*t*_ = 0.7 in this study). In the LES model, the turbulent fluxes are calculated directly.

In the wind tunnel experiments, under neutral inflow conditions, the convective fluxes were positive near the leeward wall but negative near the windward wall. The turbulent fluxes were positive in the lower part of the canyon, and a slightly positive region was observed near the windward corner. When the two were added together, the fluxes were strengthened near the leeward wall, while they canceled out near the windward wall. Under stable incoming flow, the positive area of convective fluxes increased, and the negative area decreased. The turbulent fluxes changed slightly. According to the results of the RANS flux analysis, the main reason for its failure to simulate the concentration distribution was that the convection flux was estimated incorrectly. Comparatively speaking, the overall flux distribution of LES was in good agreement with the experimental results, especially for the turbulent fluxes. However, regardless of the inflow conditions, LES overestimates the negative convective fluxes near the windward wall (i.e., the estimated negative values are more pronounced), resulting in more obvious negative values of the total fluxes than those in the experiments. The reason, as mentioned earlier, was that the main thermal of the LES was greater than that in the experiments.

In addition, the normalized air exchange rate (*ACH*) of the vertical plane (*y*/*H* = 0) was computed by integrating the vertical velocity along the canyon width at the roof level, $ACH=\int_{-0.5}^{0.5}w^{+}/U_{2H}dx$, where *w*^+^ is the mean positive vertical velocity at the roof level. Table 2 shows the normalized air exchange rate and the difference between the experiments.

**Table 2:** Normalized ACH and the difference.

CASE	Normalized *ACH*	Difference (%)
N-EXP	0.055	/
S-EXP	0.044	/
N-RANS	0.039	-29.1
S-RANS	0.021	-52.3
N-LES	0.055	0.0
S-LES	0.046	4.5

The difference is calculated according to $Difference(\%)=({ACH}_{CFD}-{ACH}_{EXP})/{ACH}_{EXP}$. In the experiment, the *ACH* was reduced by 20% under the condition of stable inflow. It is clear that RANS significantly underestimated the air exchange rate by 29.1% under neutral inflow conditions and even by 52.3% under stable conditions. The difference between the LES and wind tunnel data was no more than 5%. This also indicates that RANS is not suitable for simulating the effects of windward heating on flow and pollutants.

## 4. Conclusions

RANS and LES simulations were conducted on a reduced scale, and the results were compared with those of wind tunnel experiments to evaluate their accuracy in simulating the thermal effect (the coupling effect of temperature stratification and local thermal effect) on the flow and dispersion in an isolated bi-dimensional street canyon. The main conclusions are as follows:

- An isolated bi-dimensional canyon with an aspect ratio of 1:1 forms a typical skimming flow under neutral conditions. However, when the thermal effect is considered, especially when the windward wall is heated, the main vortex inside the canyon is broken, resulting in significant flow separation.
- The RANS method fails to predict the flow field when the windward wall is heated, leading to failure to predict the flow, temperature and concentration in the canyon regardless o whether the incoming flow temperature stratification is neutral or stable. One of the reasons is that it is a steady-state solver and cannot reproduce flow separation exactly. The other is the limitation of the Boussinesq model. It can only be used when the temperature difference is small. However, the temperature difference in this paper is 93–99, which directly leads to the failure of the RANS model. Additionally, the accuracy of the concentration field was highly dependent on the flow and turbulence field, which was restricted by steady RANS computations.
- LES remains satisfactory even when accounting for both inlet temperature stratification and local thermal effects. LES performs better than RANS in terms of both the number of regenerated vortexes and the low wind velocity in the canyon, and their results are closer to the wind tunnel results, mainly because LES can simulate flow separation. However, LES overestimates the overall temperature in the canyon because heat exchange is eliminated in LES but difficult to avoid in the experiment. Since pollutants are passively dispersed, the correct flow field is the reliable basis for the concentration field. To this end, LES performs well.
- Two suggestions for the simulation of the design thermal effect are proposed: (1) If computing resources are limited, a steady-state calculation using the Boussinesq model should be performed, except when the temperature difference in the domain is large. As a recommendation, if the margin of error is 10%, the temperature difference should be less than 27.2°C; if the margin of error is 5%, the temperature difference should be less than 13.6°C. (2) If the temperature differences are large, a transient calculation, LES, must be carried out to capture the flow separation and the turbulence caused by buoyancy.

## 5. Limitations and future work

In this paper, we solely simulate the thermal effect at a reduced scale and propose numerical simulation recommendations without conducting full-scale simulation research. As mentioned earlier, the local thermal effect significantly impacts the flow field in wind tunnel tests and numerical simulations, even altering the flow structure. However, it is found to have a negligible impact on the entire flow field during full-scale field observations. Based on comprehensive analysis, the potential reasons for this are as follows: First, two-dimensional canyons easily exhibit a multi-vortex structure in wind tunnels and numerical simulations; however, complete two-dimensional canyons do not exist in practical scenarios. Even if the block length is sufficiently long, ensuring that the flow field within the canyon remains unaffected by uneven building surfaces on both sides or edge effects of the block becomes challenging. Second, actual field measurements experience time-sensitive changes in wind speed and direction, whereas wind tunnels and numerical simulations maintain a fixed perpendicular wind direction relative to the canyon. This discrepancy can significantly affect the comparability of the results. Third, when considering thermal effects, it is possible that common Reynolds number independence criteria no longer apply since airflow depends on both the Reynolds number and Richardson number. Consequently, inconsistent results have been obtained between reduced-scale wind tunnel tests and full-scale field experiments. Fourth, limited measurement points during full-scale field observations may fail to accurately capture real-flow information at key locations. For the above reasons, full-scale simulations are not included in this paper because full-scale measurement data are very limited. However, high-quality and high-resolution full-scale field observation experiments and numerical simulations constitute one of our future research directions.
